# Immobilization of Superoxide Dismutase in Mesoporous Silica and its Applications in Strengthening the Lifespan and Healthspan of *Caenorhabditis elegans*

**DOI:** 10.3389/fbioe.2022.795620

**Published:** 2022-07-19

**Authors:** Yiling Yang, Wenbin Wang, Kefeng Liu, Jie Zhao

**Affiliations:** ^1^ Department of Ultrasound, The First Affiliated Hospital of Zhengzhou University, Zhengzhou, China; ^2^ Department of Pharmacy, The First Affiliated Hospital of Zhengzhou University, Zhengzhou, China; ^3^ Internet Medical and System Applications of National Engineering Laboratory, Zhengzhou, China

**Keywords:** ROS, SOD@MSN, *C. elegans*, lifespan, healthspan, anti-aging therapeutics

## Abstract

Senescence is a major inductive factor of aging-related diseases in connection with an accumulation of reactive oxygen species (ROS). Therefore, it is important to maintain ROS at an appropriate level to keep homeostasis in organisms. Superoxide dismutase (SOD) is a vital enzyme in defending against oxidative damage *in vivo*. Because of the defects in the direct application of SOD and SOD mimics, mounting delivery systems have been developed for the efficient applications of SOD to realize antioxidant treatment. Among these systems, mesoporous silica nanoparticles (MSNs) have been widely studied because of various advantages such as desirable stability, low toxicity, and adjustable particle sizes. Herein, SOD was immobilized on MSNs using a physical absorption strategy to construct the nanosystem SOD@MSN. The nematode *Caenorhabditis elegans* (*C. elegans*) was selected as the model organism for the subsequent antioxidant and anti-aging studies. The research results suggested the nanosystem could not only be effectively internalized by *C. elegans* but could also protect the nematode against external stress, thus extending the lifespan and healthspan of *C. elegans*. Therefore, SOD@MSN could be applied as a promising medicine in anti-aging therapeutics.

## Introduction

Senescence, which enhances susceptibility to stimulation and increases the rate of death, is a gradual physiological deterioration with age. The biological mechanism of aging is still unknown (Wurm et al., 2020; Portegijs et al., 2022). Nowadays, a number of research studies have found that reactive oxygen species (ROS) have a close relationship with aging and homeostasis in organisms (Qi et al., 2019; Yang et al., 2019). ROS, which is frequently mentioned in the field of biology and medicine, refers to oxygen-containing substances with high reactivity, specifically superoxide anion (O_2_^−^), hydrogen peroxide (H_2_O_2_), hydroxyl radical (OH˙), singlet oxygen (^1^O_2_), peroxide free radical (LOO˙), hydrogen peroxide lipid (LOOH), peroxynitro group (ONOO^−^), hypochlorous acid (HOCl), and ozone (O_3_)(Liu T. et al., 2020; Gao et al., 2021). Although ROS generated in the process of oxygen metabolism are essential for cell signaling and immune response, superfluous ROS could oxidize lipids, proteins, and nucleic acid, thus triggering various diseases such as inflammation, stroke, cancer, Alzheimer’s disease, and Parkinson’s disease (Zhao et al., 2019; Liu Y. et al., 2020; Ma et al., 2020; Yu et al., 2020). More critically, the accumulation of ROS could reduce the resistance against cellular stress including thermal and oxidative stimulation, thus accelerating the process of aging (Shi et al., 2018). Current studies aim to design drugs to improve the physiological conditions and inhibit the degeneration of age-related diseases by eliminating ROS (Wang Z. et al., 2017). *Caenorhabditis elegans* (*C. elegans*), as a kind of pseudo-coelomate nematode, has been widely applied as a model organism for aging research due to its behavioral and physiological similarity with humans when they age (Zhang et al., 2016). Notably, similar to various species, *C. elegans* accumulate oxidized proteins and have autofluorescence as they age (Meyer et al., 2010; Yee et al., 2014).

To fight against damage by ROS and maintain the oxidation level at the appropriate level, it is critical to find a well-performing antioxidant. Nowadays, various antioxidants including synthetic antioxidants, polysaccharides, and proteinaceous antioxidants have been adopted in the removal of ROS production to delay aging (Owusu-Ansah et al., 2013; Xia et al., 2013). Compared with synthetic antioxidants or polysaccharide, proteinaceous antioxidants including superoxide dismutase (SOD), catalase (CAT), and glutathione peroxidase (GPx) are more acceptable due to their excellent biocompatibility and better ability to scavenge free radicals (Wang et al., 2020; Huang et al., 2021). Previous investigators have examined the effects of overexpression of CAT genes targeting mitochondria that confer resistance to oxidation on extending the lifespan of mice. Meanwhile, results showed that reducing the mitochondrial ROS level might be important in determining mammalian longevity (Qin et al., 2020; Xi et al., 2020; Zhang et al., 2021).

SOD, as an important member of the antioxidative system, can efficiently catalyze the decomposition of superoxide radicals into hydrogen peroxide and oxygen (Udipi et al., 2000; Singh et al., 2017; Koner et al., 2021; Liu et al., 2021; Wu et al., 2021). Therefore, it has demonstrated therapeutic potential in the treatment of ROS-mediated diseases and aging. However, direct use of SOD has been hampered by poor pharmacokinetics, rapid renal clearance, degradation by proteases in the serum, and low cellular membrane–penetrating ability (Darroudi et al., 2020; Rao et al., 2021). Thus, it is necessary to develop several techniques to enhance the stability and cellular delivery ability of SOD, such as PEGylation or encapsulating proteins in vesicles (Xu et al., 2021), liposomes (Abedi Gaballu et al., 2019; Li et al., 2020), and mesoporous silica nanoparticles (MSN) (von Baeckmann et al., 2018; Wang et al., 2019). Among them, MSNs, as a kind of safe medical material approved by the United States Food and Drug Administration (FDA), have been extensively studied and applied in drug delivery due to their high specific surface area, stable pore volume, adjustable particle size, and excellent biocompatibility (Wang et al., 2016; Wen et al., 2017). In addition, the particle sizes of MSNs can be designed to be less than 100 nm, which have a larger specific surface area and are more suitable for biomedical applications (Zhang et al., 2018).

In this study, an assembly enzyme SOD@MSN was constructed by physical absorption, and the antioxidant effects of SOD@MSN were investigated. In addition, we revealed the protective effect of this assembly enzyme against stress and the anti-decrepit and life-prolonging effect of SOD@MSN on *C. elegans*. The relationship among these effects is also discussed. The mechanism of action is also expounded, including the assembly’s antioxidant activity and the effects on stress-related proteins and genes.

## Materials and Methods

### Materials

Cetyltrimethylammonium bromide (CTAB), tetraethyl orthosilicate (TEOS), 1,2-Bis(triethoxysilyl) ethane (BTEE), triethanolamine (TEA), and sodium salicylate (NaSal) were purchased from Sigma-Aldrich. Hydrochloric acid (HCl), ethanol, and methanol were purchased from Aladdin. In addition, 5-fluoro-2-deoxyuracil (FUDR) and phenylmethyl sulfonyl chloride (PMSF) were purchased from Sigma Corporation (United States); SOD enzyme was purchased from Bairdi Company (Beijing, China). TRNzol Universal Reagent was purchased from TIANGEN (Beijing, China); PrimeScript™ RT kit and SYBR® Premix Ex Taq™ kit were purchased from TAKARA Company (Dalian, China). All chemicals were used as received without purification.

### Synthesis of Mesoporous Silica Nanoparticles and Superoxide Dismutase@Mesoporous Silica Nanoparticles

MSN and SOD@MSN were achieved through a one-pot synthesis using the cationic surfactant CTAB, NaSal as the structure-directing agent, TEOS and BTEE as silica sources, and TEA as a catalyst. A typical synthesis of MSN was carried out as follows: First, 0.05 g TEA was dissolved in 15 ml of water and stirred gently at 80°C in an oil bath under a magnetic stirring for 1 h. Then, 360 mg CTAB and 150 mg NaSal were added to the aforementioned solution and then stirring was continued for another 1 h. A mixture of 1.8 ml TEOS and 1.5 ml BTEE was added to the NaSal–TEA solution with or without SOD with gentle stirring for 10 h. The products were collected by centrifugation and washed five times with ethanol. Then, the obtained products were extracted with HCl and methanol solution at 60°C for 5 h to eliminate the template, and then dried in a vacuum at room temperature for 12 h.

### Synthesis of FITC-Superoxide Dismutase@Mesoporous Silica Nanoparticles

20 mg SOD and 10 mg FITC were dissolved in 50 ml phosphate buffer (50 mM, pH 8.0), and the mixture was stirred at 180 rpm at room temperature for 24 h. The sample was then washed with distilled water using Amicon® ultra-15 until no absorption value could be detected in the ultrafiltration supernatant at 488 nm. Finally, FITC-SOD was obtained by freeze-drying and used as raw material. The synthetic method of FITC-SOD@MSN was the same as the aforementioned method.

### Determination of the Relative Enzymatic Activity

The load of lipase in SOD@MSN was calculated by formula A-A_1_/B×100%, where A, A_1_, and B, respectively, represent the weight of input SOD, the weight of SOD in the supernatant after reaction, and the total weight of SOD@MSN synthesized. The quality of lipase in the supernatant was determined by the BCA protein quantification kit (Beyotime, Nanjing, China). The enzymatic activity was assessed by the pyrogallol autoxidation assay. In detail, 240 μl enzyme solutions (1 mg/ml) was added to 750 μl Tris-HCL (pH 8.2) and incubated for 20 min. 10 μl pyrogallol solution (50 mM) was then mixed into the enzyme solutions and the absorbance at 325 nm was measured for 1 min by the UV-2700 spectrometer (Shimadzu, Kyoto, Japan).

### Worm Strains and Culture

*C. elegans* was cultured in a nematode growth medium (NGM) covered with *E. coli* OP50 at 20°C. To obtain a synchronous nematode, we cultured gravid worms for 5 h and then the eggs were collected by treating the eggs and adult *C. elegans* with alkaline hypochlorite solution (NaOH: NaClO: H_2_O = 2:1:1); we continued to culture in the NGM medium for about 12 h. The worms of the control group were cultured in NGM plates coated with 5-fluoro-20-deoxyuridine (FUDR, 100 μM) dissolved in *E. coli* OP50, and FUDR was used to prevent progeny growth. The worms in the treatment groups were grown in NGM medium coated with SOD@MSN, free SOD or MSN, and FUDR dissolved in *E. coli* OP50.

### Uptake of Superoxide Dismutase@Mesoporous Silica Nanoparticles by *C. elegans*

In order to observe the effect of SOD and SOD@MSN uptake by nematodes more intuitively, we labeled SOD with FITC, and then assembled FITC-SOD onto MSN. The L4-stage nematodes were transferred to NGM culture plates containing FITC-SOD, FTIC-SOD@MSN, or MSN for 2 h. The nematodes were then washed from the NGM culture plate with M9 buffer and centrifuged (2000 rpm, 1 min) for collection. Then, the nematodes were anesthetized with levamisole and transferred to slides containing 3% agarose for fixation. Finally, the nematodes were observed and photographed under a fluorescence microscope.

### *In vivo* Retention Time of Superoxide Dismutase@Mesoporous Silica Nanoparticles in *C. elegans*

*C. elegans* at the L4 stage were cultured in NGM culture plates containing FITC-SOD or FITC-SOD@MSN for 6 h and then transferred to fresh NGM culture plates for further culture. At 0.5, 1, 2, 4, 6, 8, 10, 12, 14, 16, and 18 h, three nematodes were removed from the two groups of culture plates, respectively. After levamisole anesthesia, the nematodes were fixed to a glass slide containing 3% agarose. Fluorescence microscopy was used to observe the changes of FITC fluorescence in nematodes at different times, and photographs were taken until the fluorescence in nematodes of the FITC-SOD group was completely quenched. The fluorescence intensity *in vivo* was quantified by ImageJ software.

### Lifespan Assay

*C. elegans* was used in the lifespan assay at least three times independently at 20°C. The assay started when the synchronous worms were grown to the end stage of Level four, which was set as day 1. There were 80 worms in each of the groups, and the fresh treatment plates were replaced every 2 days. Tested *C. elegans* were considered to be dead when they failed to respond under the stimulus of a platinum wire.

### Healthspan Assay

Oil Red O stain which has been widely regarded as a dye for lipid deposits was used for the detection of the fat levels in *C. elegans*. Forty synchronous worms per group were raised to the L4 level, which was regarded as day 0. Furthermore, the worms were transferred to plates with or without SOD formulations and continued to culture for 5 days. Then, the nematodes of various groups were washed with PBS three times and fixed with 1% paraformaldehyde for 20 min. Subsequently, the *C. elegans* was subjected to three repeated freezing and thawing between −80 and 40°C, after which the worms were treated with 60% isopropanol to dehydration. Finally, 60% Oil-Red-O staining solution was added and stained for 20 min followed by washing with PBS two times and photograph collection by fluorescence microscopy.

The spontaneous fluorescence was then detected as a signal of aging. Fifty animals per group were cultured from L1 level on the NGM plates with or without drugs. After ten days, the worms were washed with M9 buffer three times and anesthetized with levamisole. Afterward, 3% agarose pads were used to immobilize the worms, and then the spontaneous fluorescence was visualized through fluorescence microscopy and quantified by ImageJ. All experiments were performed in triplicate.

### Assessment of Stress Resistance

The age-synchronized *C. elegans* were first cultured on NGM plates with or without samples at 20°C for 2 days, and then subjected to plates with juglone (500 μM). The survival worms were recorded every hour over 12 h.

The selected 2-day-old nematodes were subjected to the same treatment as described above for 48 h and then incubated at 35°C, which was considered a thermal stimulation. The number of surviving animals was recorded over 8 h, and all experiments were conducted at least three times.

### Assessment of Intracellular Reactive Oxygen Species Levels

The worms that had just grown up into adults were selected for the experiment. For detection under normal conditions, the animals were treated with various SOD formulations for 2 days. For detection under oxidative environment, the worms were first subjected to juglone (300 μM) before treatment with samples for 2 days. Subsequently, the animals were washed and collected by M9 buffer in suspension. Finally, ROS fluorochrome H2DCF-DA and the suspension of worms were pipetted into 96-well plates, respectively, with the final concentration of H2DCF-DA at 50 μM. The fluorescence was detected every 30 min over 150 min at 20°C by a microplate fluorescence reader (Olympus, Tokyo, Japan) with excitation/emission wavelengths at 485 and 538 nm, respectively. The fluorescence intensity at 30 min without drugs’ treatment was set as 100%.

### Assessment of Intracellular Superoxide Dismutase Activity

N2 worms were cultured on NGM plates with or without drugs’ treatment for one week. During this process, the worms were transferred to fresh plates every 2 days. Afterward, the total protein in nematodes was extracted. Briefly, the animals were washed down to the EP tubes using M9 buffer and centrifuged at 1,000 rpm for 2 min to remove M9. Subsequently, 150 μl RIPA with 1.5 μl PMSF was added to re-suspend the worms, and then the samples were subjected to three fast freeze–thaw cycles. Finally, the total protein was collected by centrifuging at 8,000 rpm for 1 min, and the supernatants were used to detect the SOD enzymatic activity by Total Superoxide Dismutase Assay Kit with WST-8 (Beyotime). To normalize the enzymatic activity, the protein concentration was measured by BCA assay kit (Beyotime).

### Quantitative Real-Time PCR

After treatment with or without SOD formulations for 2 days, total RNAs of the worms were extracted with Trizol Reagent, and cDNA was generated by PrimeScript® RT reagent Kit. Afterward, the mRNA expressions of *cdc-42*, *daf-16*, *daf-2*, *sod-3*, *hsp-16.2*, and *skn-1* were determined on a 7500 Real-Time PCR System (Applied Biosystems, Foster City, CA) using SYBR green as the detection reagent. The data were calculated using the 2^−ΔΔCT^ method, and *cdc-42* was used as a control gene for normalization. The primers were as follows:

*daf-16* forward: 5′-TTT​CCG​TCC​CCG​AAC​TCA​A-3′;
  reverse: 5′-ATT​C-GCC​AAC​CCA​TGA​TGG-3′;
*sod-3* forward: 5′-AGC​ATC​ATG​C-CAC​CTA​CGT​GA-3′;
  reverse: 5′-CAC​CAC​CAT​TGA​ATT​TCA​GCG-3′;
*daf-2* forward: 5′-GGC​CGT​AGG​ACG​TTT​A-TTT​G-3′;
  reverse: 5′-TTC​CAC​AGT​GAA​GAA​GCC​TGG-3′;
*hsp-16.2* forward: 5′-C-GTC​GAA​GAG​AAT​ACT​GCT​GAA-3′;
  reverse: 5′-TGC​AGC​GAA​CAT​AAC​T-GTA​TAT​TTA​G-3′;
*skn-1* forward: 5′-AGT​GTC​GGC​GTT​C-CAG​ATT​TC-3′;
  reverse: 5′-GTC​GAC​GAA​TCT​TGC​GAA​TCA-3′;
*cdc-42* forward: 5′-CTG​CTG​GAC​AGG​AAG​ATT​ACG-3′;
  reverse: 5′-CTCGGACATTCTCGA ATGAAG-3′.

## Results and Discussion

### Synthesis and Electron Microscopic Analysis of Superoxide Dismutase@Mesoporous Silica Nanoparticles

**FIGURE 1:**
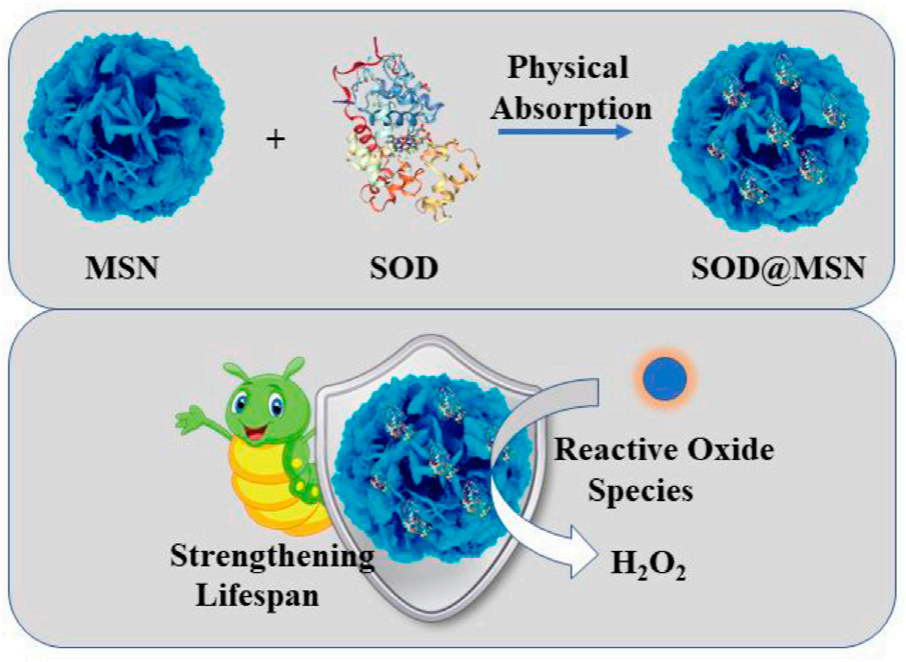
The detailed construction process of SOD@MSN and its application in strengthening the lifespan of *C. elegans*.

**FIGURE 2:**
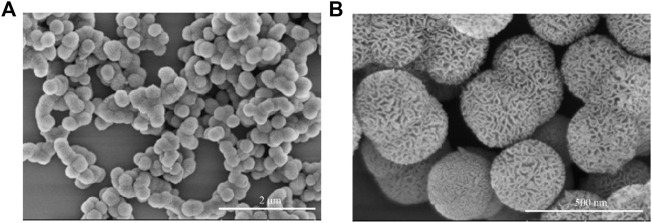
The morphology of SOD@MSN detected by SEM with the scale bar 2 um and 500 nm.

**FIGURE 3:**
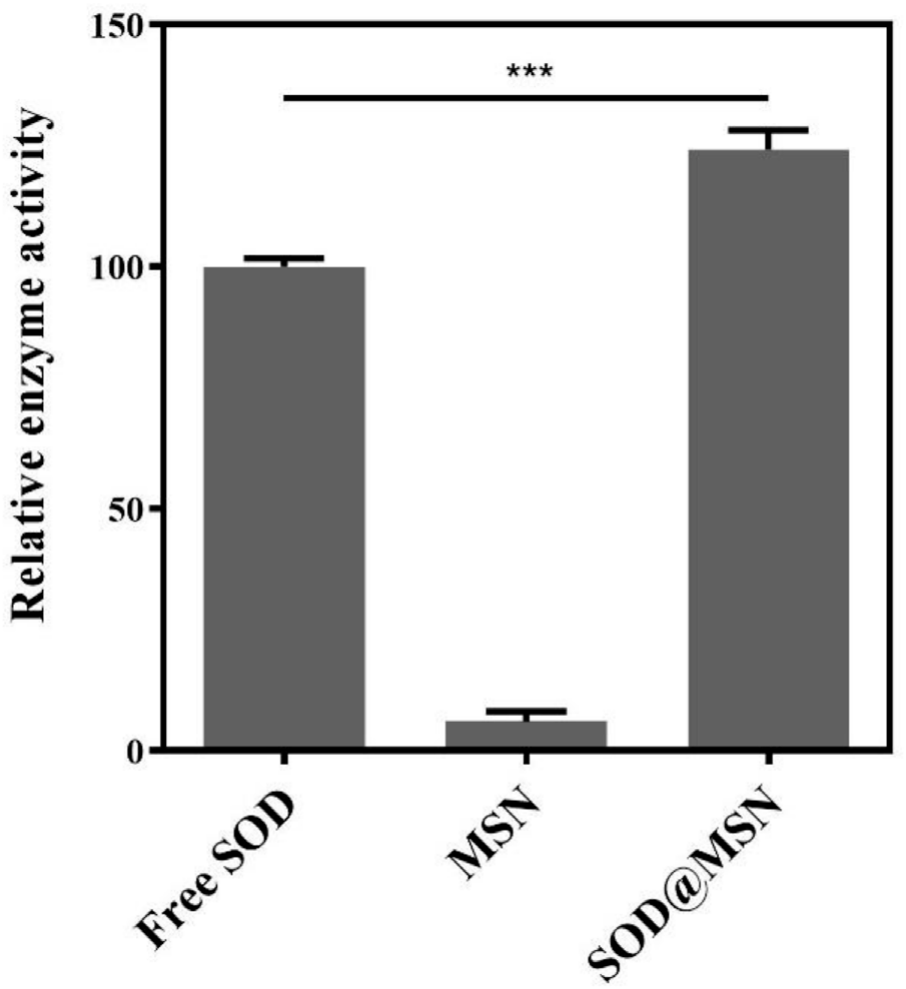
Enzymatic activity of SOD@MSN and free SOD detected through pyrogallol autoxidation assay.

First, we synthesized SOD@MSN with various ligand ratios (TEOS: BTEE from 0.8 to 1.4) and found that only the given ligand ratio and reaction conditions could obtain the homogeneous morphology of SOD@MSN (Figures 1, 2 and Supplementary Figure S1). In addition, SOD content was 10.36 wt% in SOD@MSN determined by BCA quantitative method. SOD@MSN at the given dose possessed the best SOD activity under the same SOD content (Supplementary Figure S2) and the enzymatic activity was concentration-dependent (Supplementary Figure S3). The nanosystem was constructed through physical absorption (Wang P. et al., 2017). The morphology of nanomaterial SOD@MSN was characterized by SEM (Figure 2), which evidently illustrated that SOD@MSN had a regular sphericity and suitable particle size to be intake by *C. elegans*. The zeta potential distribution of SOD@MSN was also determined, which suggested that the surface charge were +35.5 and +39.0 mV in water and PBS solutions, respectively (Supplementary Figure S4). Further, the enzymatic activity of SOD@MSN was detected by pyrogallol autoxidation assay, which suggested that the SOD activity was significantly increased by assembling into MSN (Figure 3).

### Uptake Efficacy of Superoxide Dismutase@Mesoporous Silica Nanoparticles by *C. elegans* and *in vivo* Stability

**FIGURE 4:**
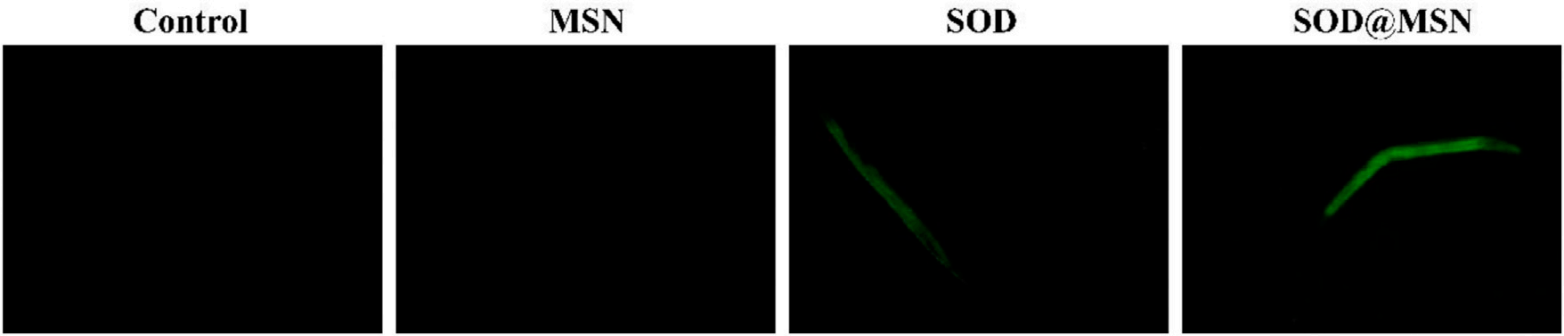
The uptake of SOD and SOD@MSN by *C. elegans* detected by a fluorescence microscope.

**FIGURE 5:**
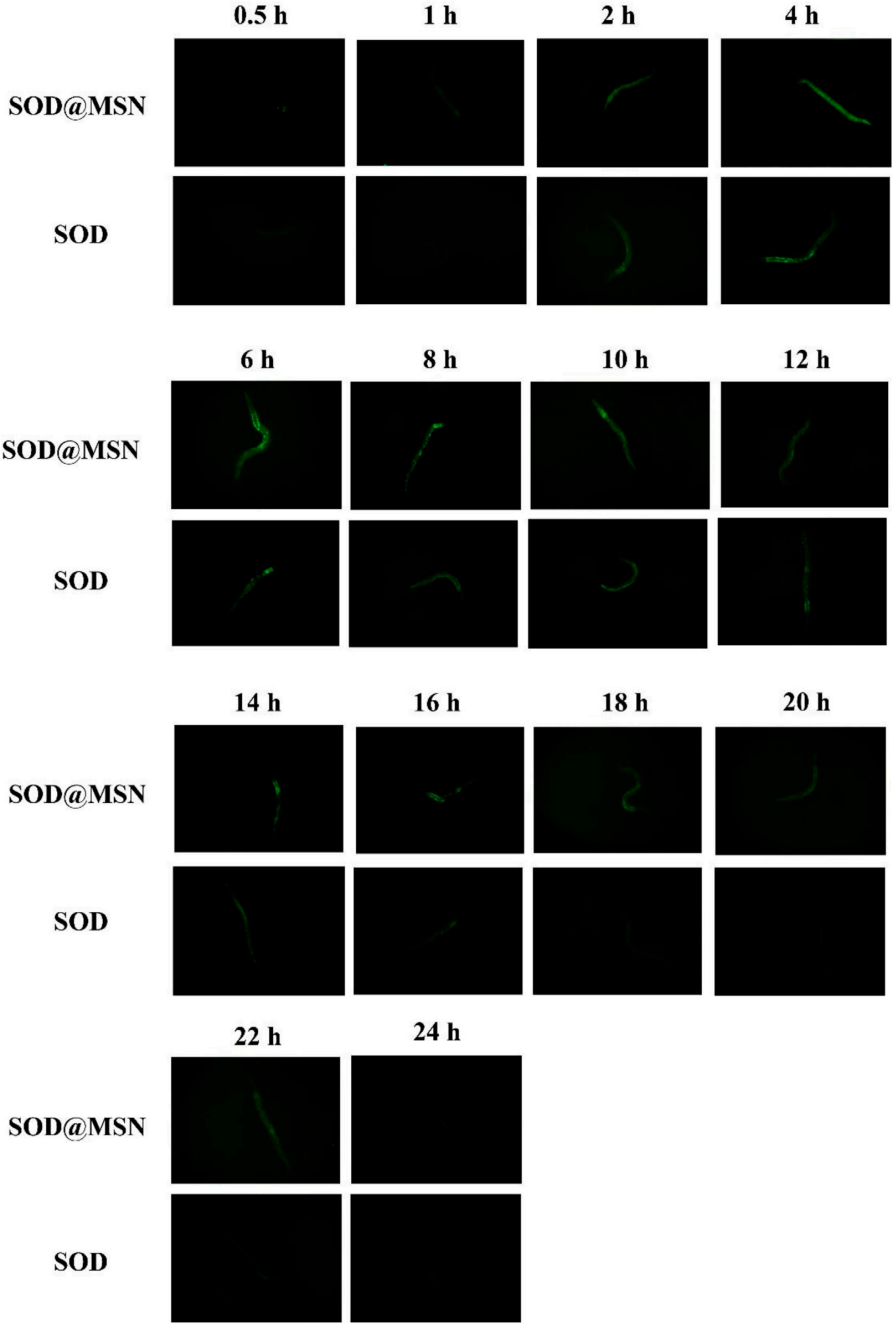
The fluorescence changes of FITC labeled SOD and SOD@MSN in *C. elegans* detected by a fluorescence microscope.

**FIGURE 6:**
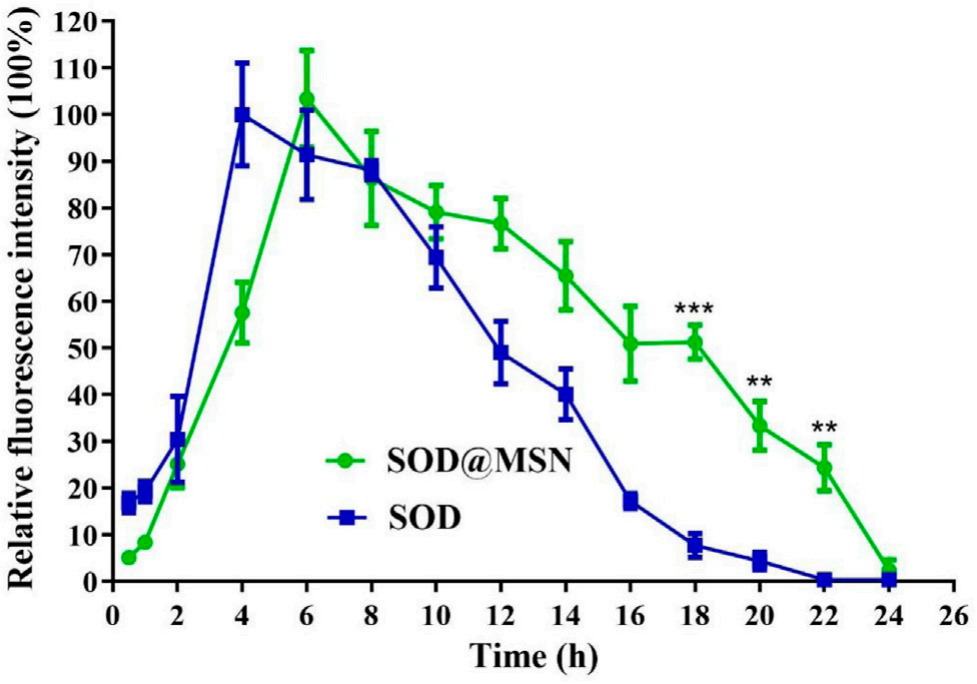
Quantitative analysis of the fluorescence changes of FITC labeled SOD and SOD@MSN in *C. elegans*.

**FIGURE 7:**
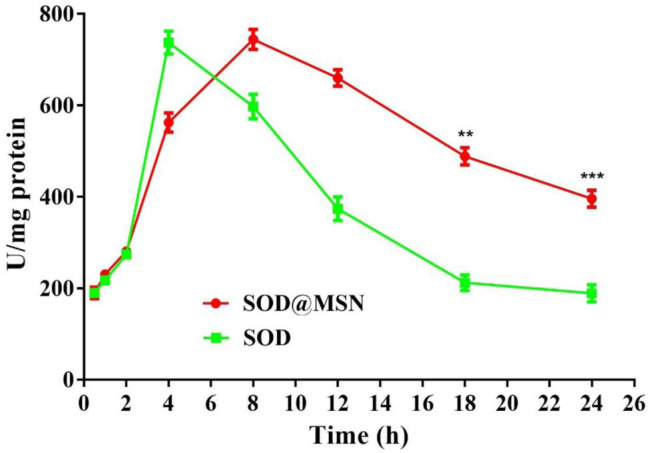
Quantitative analysis of the SOD enzymatic activity at various time points after SOD@MSN or free SOD treatment.

Effective entry of assembling enzymes into *C. elegans* is the prerequisite for them to exert an antioxidant role. Therefore, we first studied the uptake capacity of *C. elegans* to SOD@MSN and its stability in worms. The results were shown in Figure 4. Both FITC-SOD@MSN- and FITC-SOD–treated nematodes showed green fluorescence, indicating that SOD@MSN and free SOD could be absorbed by nematodes. Then, *C. elegans* were treated with FITC-labeled free enzymes or assembled enzymes for 6 h, and then transferred to NGM culture plates without fluorescence. The quenching of FITC fluorescence in nematodes was observed and recorded during the whole culture process, and the degradation rate of FITC in nematodes was investigated. Figure 5 showed that the fluorescence of nematodes in both FITC-SOD@MSN and FITC-SOD groups underwent a change from weak to strong and then to weak. Fluorescence quantitative results obtained by ImageJ software (Figure 6) showed that the fluorescence of *C. elegans* treated with FITC-SOD@MSN reached the maximum at the sixth hour, while that of nematode treated with FITC-SOD reached the maximum at the fourth hour, indicating that the absorption of nematode to SOD@MSN was slightly slower than that of free SOD. However, the fluorescence of FITC-SOD@MSN treated nematodes was still obvious at the eighteenth hour, while the fluorescence of FITC-SOD–treated nematodes was almost completely quenched. At the same time, the fluorescence intensity of the FITC-SOD@MSN–treated group was higher than that of free SOD in the range of 12 to 22 h. In addition, the sizes of SOD@MSN in *C. elegans* were detected in a time-dependent manner, which suggested that the size of the nanoparticle in the body of *C. elegans* remained around 300 nm (Supplementary Figure S5). Furthermore, the SOD enzymatic activity detection at various time points after SOD@MSN or free SOD treatment was also carried out, which suggested that the SOD activity attenuated more slowly after SOD@MSN treatment (Figure 7). The aforementioned results indicated that SOD@MSN has better stability than free SOD in *C. elegans* and is expected to achieve a better therapeutic effect.

### Effect of Superoxide Dismutase@Mesoporous Silica Nanoparticles on the Lifespan of *C. elegans*

**FIGURE 8:**
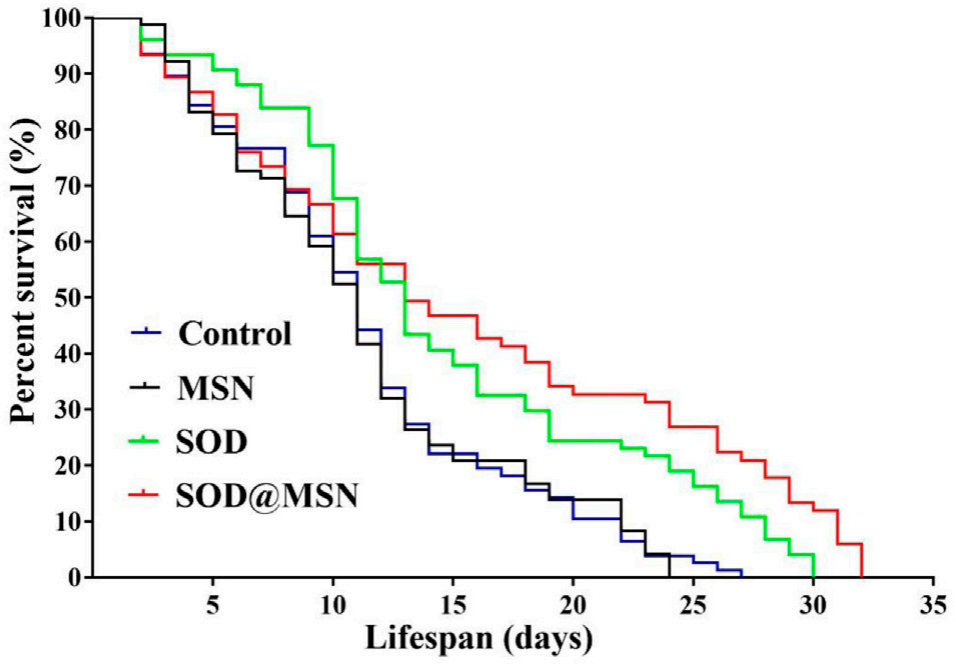
The effect of SOD@MSN on lifespans of *C. elegans*.

In order to determine the longevity effect of SOD@MSN on wild-type *C. elegans*, lifespan research was carried out, and 20°C was selected as the experimental temperature because it was optimal for *C. elegans.* As can be clearly illustrated in Figure 8 and Table 1, SOD@MSN and free SOD had the effect of prolonging the lifespan of worms with the mean lifespan increased from 10.23 to 16.12 and 14.04, respectively. In addition, the maximum lifespan of *C. elegans* also had a marked improvement with the treatment of SOD@MSN and free SOD. However, there was no effect after treating with MSN. The aforementioned results suggested that SOD@MSN and SOD had a satisfactory life-prolonging effect on wild-type *C. elegans.*

**TABLE 1:** Statistical table of the influence of SOD@MSN on the mean life span, mean life change, and maximum life span of *C. elegans*.

Genetype	Food	Treatment (20°C)	Total (N)	Mean		Maximum lifespan	Change in mean lifespan	Log-rank test	
				Lifespan	SE		(%)	X^2^	P
Wild-type	OP50	Control	80	10.23	0.424	26			
Wild-type	OP50	SOD@MSN	75	16.12	1.678	33	36.49	6.214	0.0045
Wild-type	OP50	SOD	75	14.04	0.862	31	25.83	3.456	0.0483
Wild-type	OP50	MSN	80	9.78	0.810	23	−3.42	0.0382	0.8016

### Effect of Superoxide Dismutase@Mesoporous Silica Nanoparticles on the Healthspan of *C. elegans*

**FIGURE 9:**
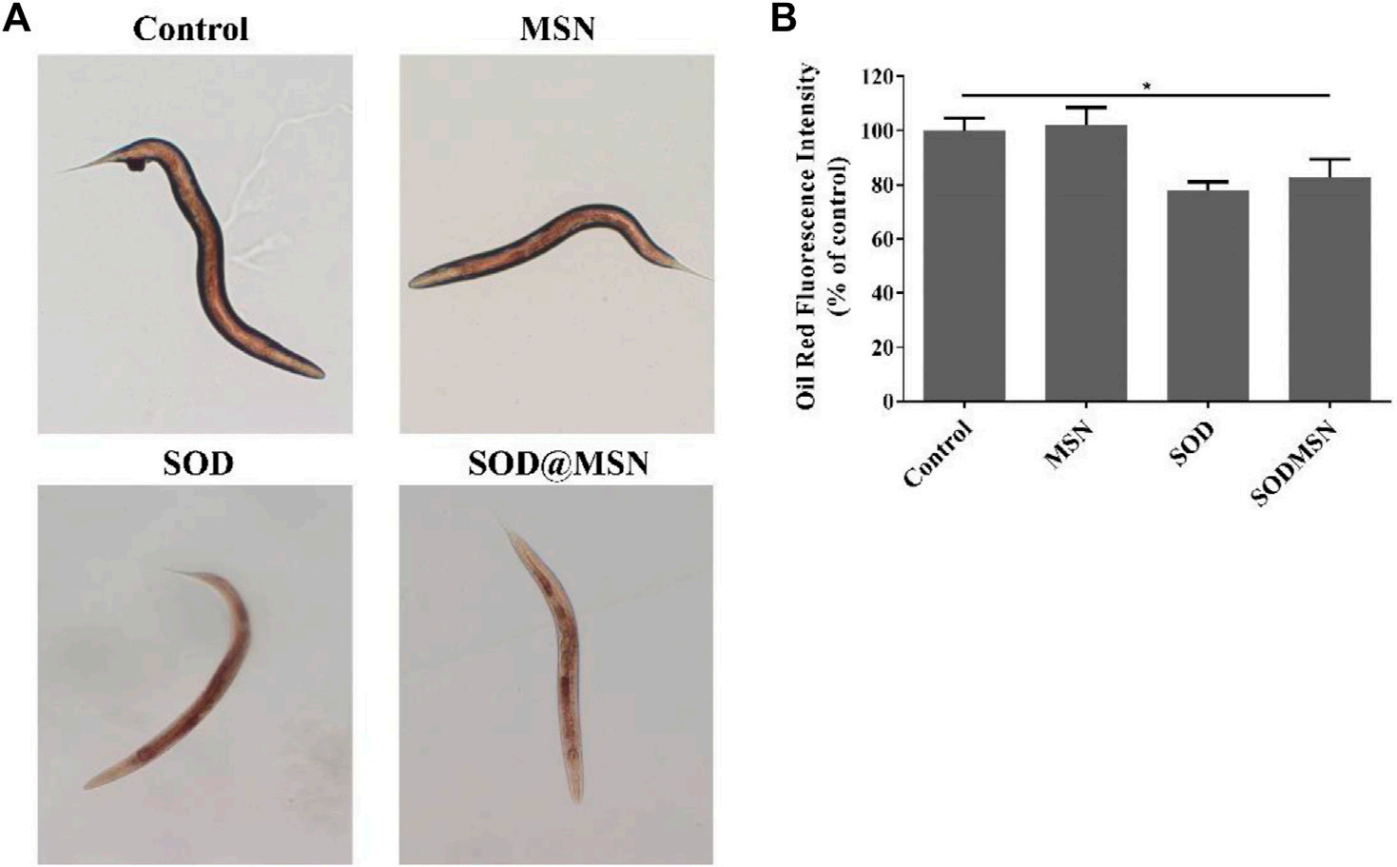
Oil Red O staining analysis **(A)** and the corresponding quantitative results **(B)** of *C. elegans* treated with SOD@MSN.

**FIGURE 10:**
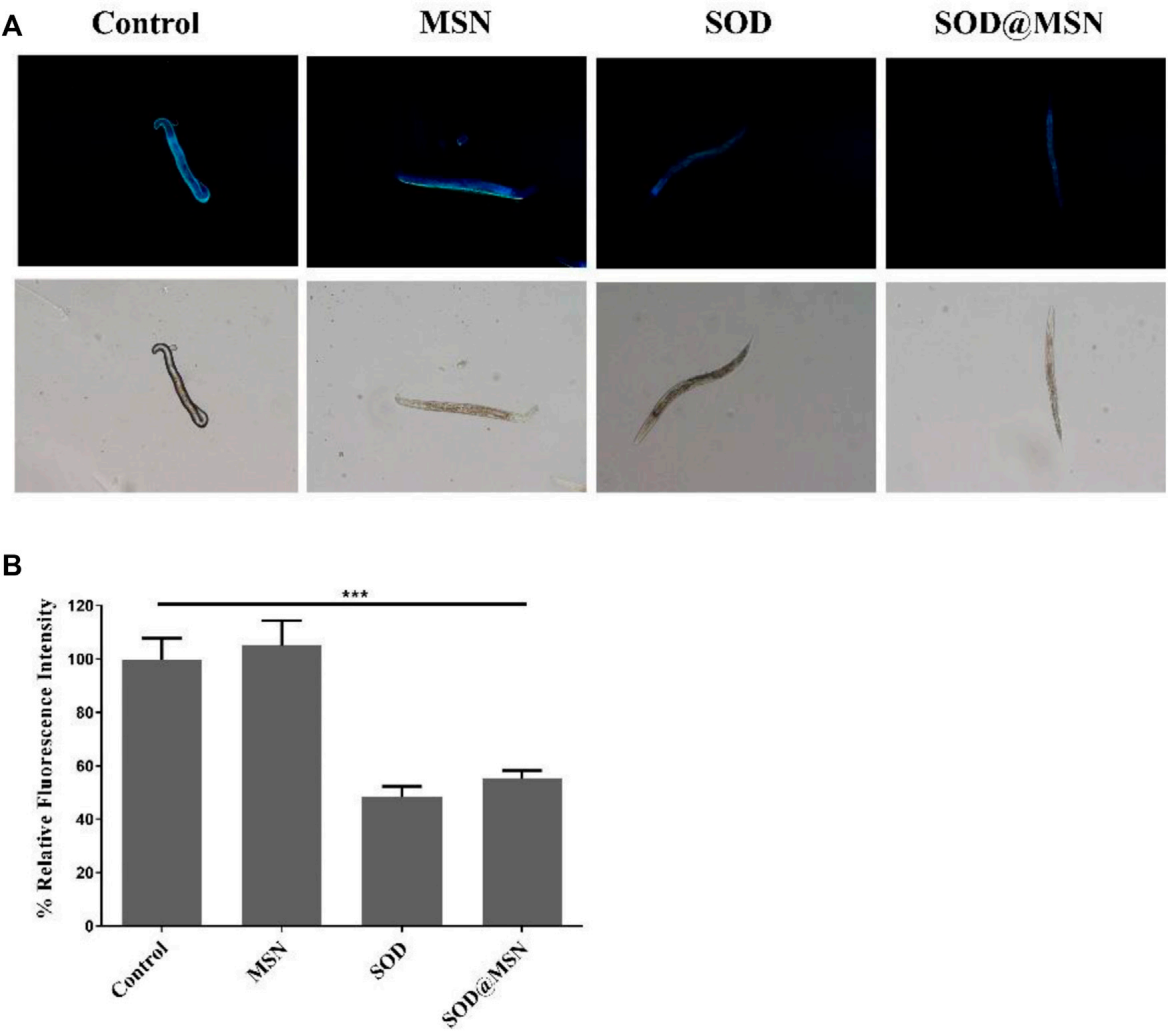
*In vivo* spontaneous fluorescence analysis of *C. elegans* treated with SOD@MSN **(A)** and the quantitative results **(B)**.

The longevity effect of SOD@MSN reminded us to study if it can improve the healthspan of *C. elegans*. First, Oil Red O was used to stain lipid deposits, which is a marker to reflect the body’s healthy levels of nematodes. As illustrated in Figure 9A, the fluorescence intensity in worms reduced significantly after treatment with SOD formulations compared with the control group. When quantified by ImageJ software, we found that the contents of lipid in N2 were reduced by 17.3% for SOD@MSN and 22% for SOD, respectively (Figure 9B). Furthermore, in contrast to the control group, the groups treated with SOD@MSN or free SOD released less blue fluorescence, as evidenced by the fluorescence microscope (Figure 10A). Quantification of the fluorescence revealed that about 48% and 55% of spontaneous fluorescence intensity was reduced in N2 with the treatment of drugs (Figure 10B). The aforementioned findings demonstrated that SOD@MSN could prominently improve the healthy levels of worms.

### Effect of Superoxide Dismutase@Mesoporous Silica Nanoparticles on the Stress Tolerance of *C. elegans*

**FIGURE 11:**
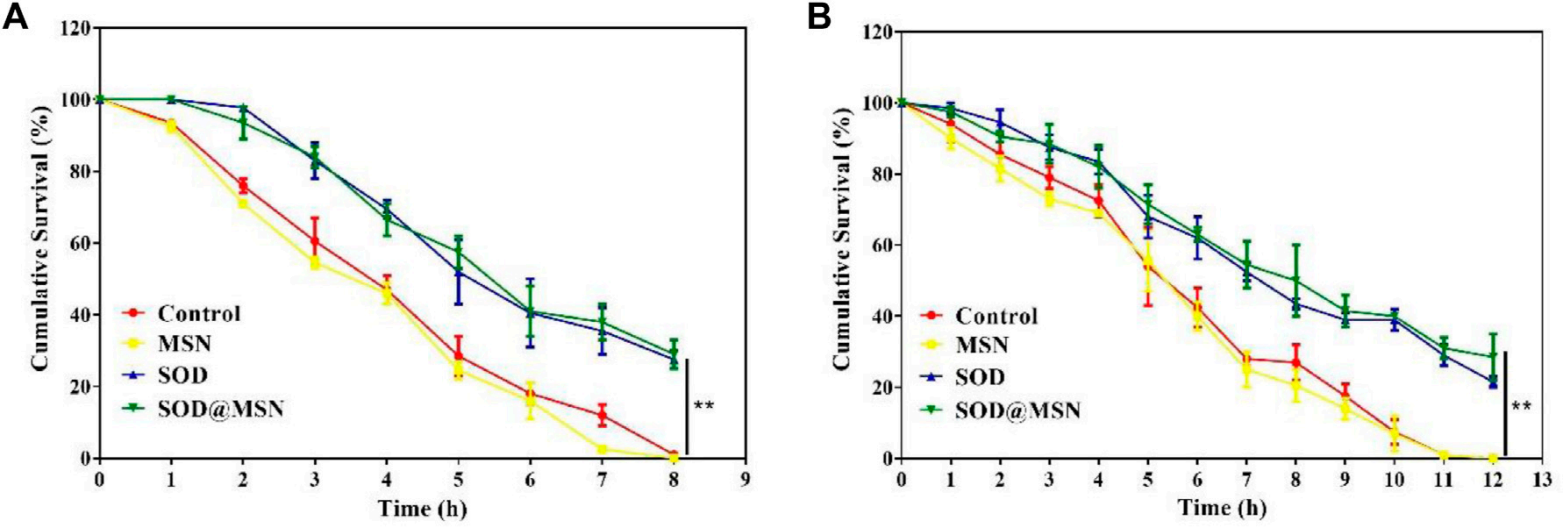
Survival rate of SOD@MSN-treated *C. elegans* in 35°C conditions **(A)** and oxidation environment **(B)** induced by juglone.

To determine if SOD@MSN could improve the stress tolerance ability of *C. elegans*, two different varieties of stress conditions were tested. First, thermo-tolerance ability of N2 was investigated using 35°C as thermal stimulation. The data suggested that SOD@MSN treatment significantly prolonged the survival time of N2 (Figure 11A). Similar results could also be found in the oxidative stress environment, in which juglone was used as the source of the oxidative stimulus. Juglone, as a kind of pro-oxidant, can produce superoxide anions in the presence of NAD(P)H and oxygen. Obviously, N2 animals treated with SOD@MSN or free SOD exhibited improved resistance against juglone, as can be proved by the increased survival rate after being pre-treated with SOD formulations (Figure 11B). The aforementioned results suggested that the worm’s ability to resist heat and oxidative stress was elevated after pre-treatment with SOD@MSN, as can be evidenced by the increased survival numbers *C. elegans*.

### Effect of Superoxide Dismutase@Mesoporous Silica Nanoparticles on the Intracellular Superoxide Dismutase Enzymatic Activity and Reactive Oxygen Species Levels

**FIGURE 12:**
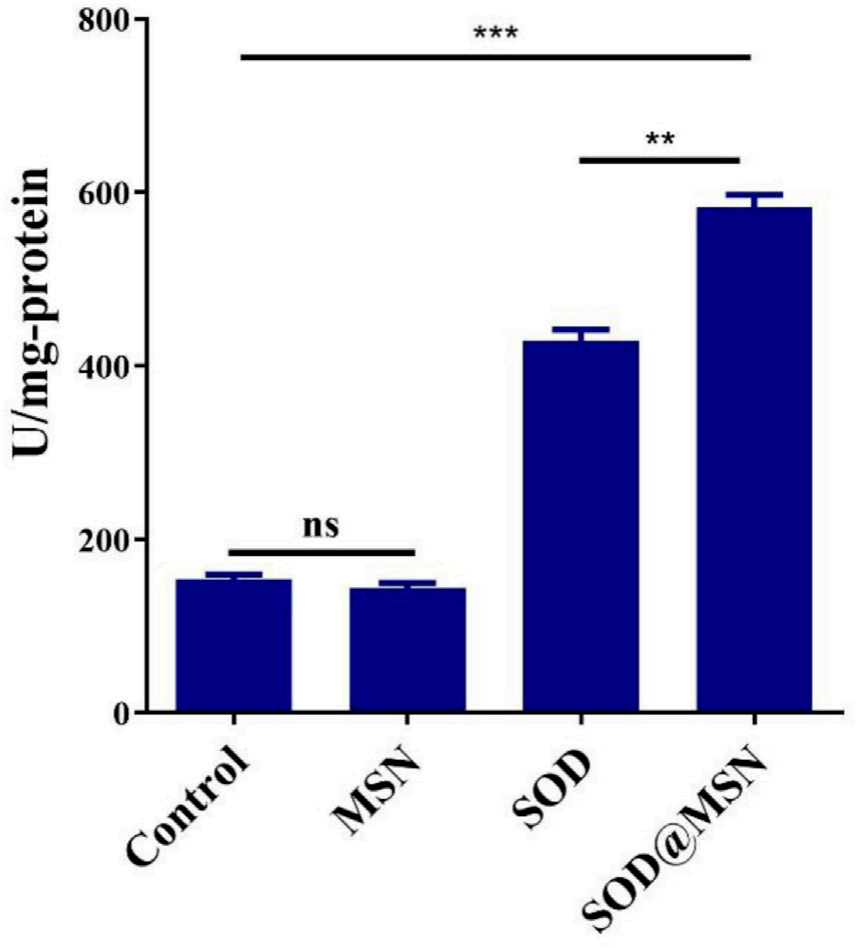
The intracellular SOD enzymatic activity after treatment.

To investigate the mechanism behind the effect of longevity and stress tolerance of *C. elegans* by SOD@MSN, the intracellular SOD enzymatic activity and ROS levels were detected. As obviously illustrated in Figure 12, the specific activity of SOD increased significantly after SOD@MSN treatment (compared with the control, *p* < 0.001; compared with the SOD group, *p* < 0.01). Furthermore, we found that the SOD activity was dramatically improved after SOD@MSN treatment than free SOD, which might be attributed to the longer staying time and higher enzyme activity in the case of the same dose of SOD.

**FIGURE 13:**
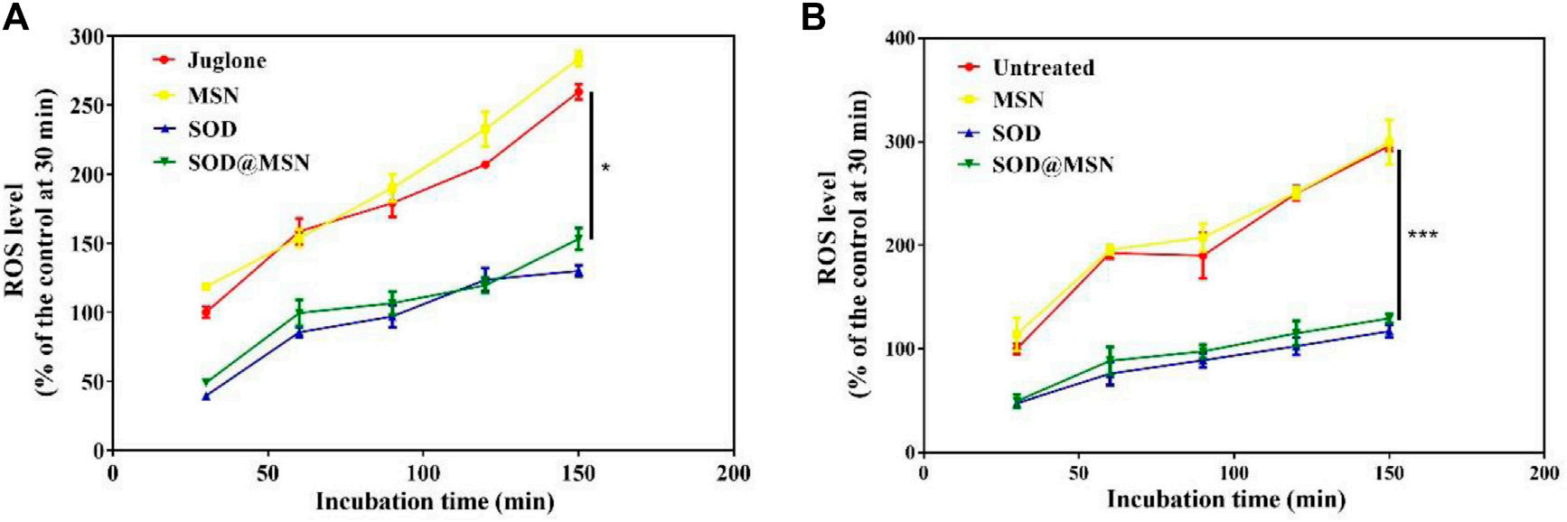
Effects of SOD@MSN on ROS content in *C. elegans* with **(A)** or without **(B)** juglone stimulation.

Afterward, the ROS levels in *C. elegans* were determined under a regular culture medium, which revealed that SOD@MSN and free SOD obviously inhibited the production of intracellular ROS (Figure 13B, compared with the control, *p* < 0.001). Furthermore, juglone was used as an exogenous ROS irritant after treatment with SOD formulations. From Figure 13A we could find that the ROS levels in worms decreased significantly after being pre-treated with SOD@MSN or free SOD (*p* < 0.05), compared with the solvent-treated control N2 *C. elegans*. The aforementioned results suggested that SOD@MSN prolonged the lifespan and strengthened the ability to resist the environmental stress by increasing the levels of antioxidant enzymes and reducing the ROS content in the body of *C. elegans.*

### Effect of Superoxide Dismutase@Mesoporous Silica Nanoparticles on the mRNA Expression of Genes Relevant to Aging and Stress Resistance

**FIGURE 14:**
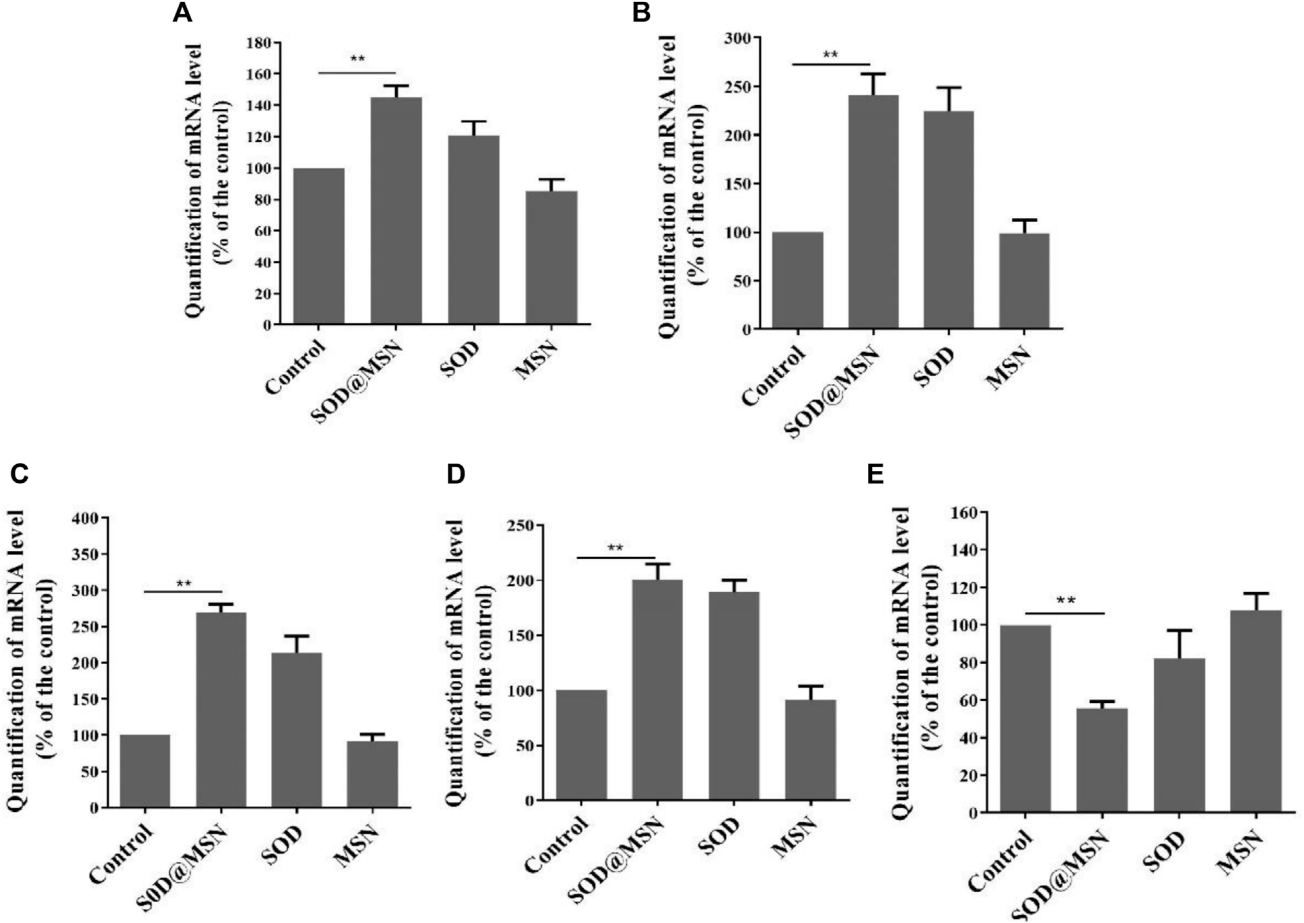
Effects of SOD@MSN on aging-related gene expression levels in *C. elegans*: *daf-16* **(A)**, *hsp-16.2* **(B)**, *sod-3* **(C)**, *skn-1* **(D)**, and *daf-2* **(E)**.

In order to investigate if the longevity and elevated stress tolerance of *C. elegans.* After SOD@MSN treatment was the result of the regulation of stress–response genes. Quantitative real-time PCR was therefore carried out to detect the expression levels of genes, including *daf-2*, *daf-16*, *sod-3*, *skn-1*, and *hsp-16.2*. DAF-16 is responsible for both lifespan and stress response, and SOD-3 and HSP-16.2 are the downstream effectors of DAF-16. In addition, SKN-1 is another factor which plays a positive role in the regulation of lifespan and stress response. However, DAF-2 acts as an inhibitor of DAF-16 by phosphorylating DAF-16 and restricting its internalization into the cell nucleus. The results are shown in Figure 14, which reveal that SOD@MSN could significantly increase the expression of *daf-16* (*p* < 0.01), *sod-3* (*p* < 0.01), *hsp-16.2* (*p* < 0.01), and *skn-1* (*p* < 0.05) and reduce the expression of *daf-2* (*p* < 0.01). These results illustrated that the effect of SOD@MSN on the lifespan and stress tolerance was the result of regulation on the aging-related genes discussed earlier.

## Conclusion

SOD was successfully immobilized on MSNs to develop an SOD@MSN nanoplatform with desirable morphology and particle size. The research results suggested that SOD@MSN could be internalized by *C. elegans* effectively and kept stable for a longer time than with free SOD. In addition, SOD@MSN significantly prolonged the lifespan of *C. elegans* and extended the healthspan and lifespan during heat or/and oxidative stress. Further studies illustrated that the obvious longevity-extending effects of SOD@MSN on *C. elegans* were attributed to its free radical–scavenging capabilities. In addition, real-time PCR results demonstrated that the up-regulation of aging-associated genes, such as *daf-16*, *sod-3*, *hsp-16.2*, and *skn-1*, also contributed to the stress-resistance effect of SOD@MSN. These results indicated that the nanoplatform could protect against environmental stress and thus improve the healthspan and survival status, ultimately benefiting the lifespan of *C. elegans*.

## Data Availability

The original contributions presented in the study are included in the article/Supplementary Material; further inquiries can be directed to the corresponding authors.
