# Supplementary material for: Immobilization of Superoxide Dismutase in Mesoporous Silica and its Applications in Strengthening the Lifespan and Healthspan of Caenorhabditis elegans
Source: Front Bioeng Biotechnol. 2022 Jul 19;10:795620. doi: 10.3389/fbioe.2022.795620 (PMC9343863; doi:10.3389/fbioe.2022.795620)
Supplement: Supplementary file 1 [file DataSheet1.docx]

**Supporting Information**

**Immobilization of Superoxide Dismutase in Mesoporous Silica and its Applications in Strengthening the Lifespan and Healthspan of *Caenorhabditis elegans*.**

Yiling Yang ^1,*^, Wenbin Wang ^2^, Kefeng Liu [^2^](mailto:Liud;liukefeng-num.1@163.com), Jie Zhao ^2,3,*^,

^1^ Department of Ultrasound, The First Affiliated Hospital of Zhengzhou University, Zhengzhou,Henan, 450052, China

^2^ Department of Pharmacy, The First Affiliated Hospital of Zhengzhou University, Zhengzhou, Henan, 450052,China

^3^ Internet Medical and System Applications of National Engineering Laboratory, Zhengzhou, Henan, 450052, China

***^*^****Corresponding author.*

E-mail: [*fccyangyl@zzu.edu.cn*](mailto:fccyangyl@zzu.edu.cn) *(Yiling, Yang)*

[*zhaojiezzu@163.com*](mailto:zhaojiezzu@163.com) (Jie Zhao)

**
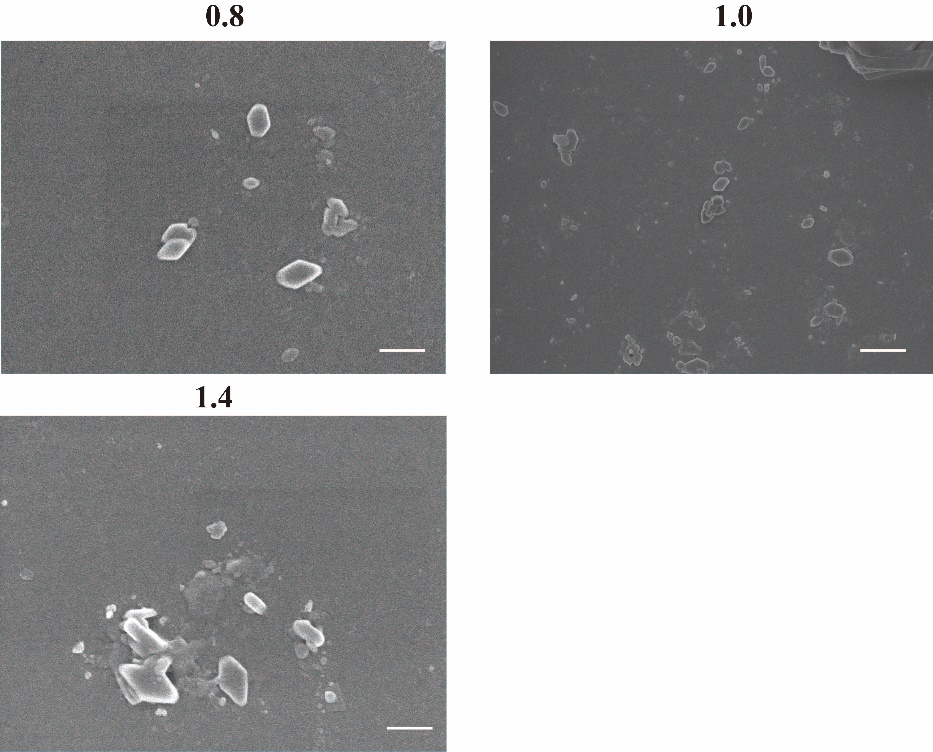

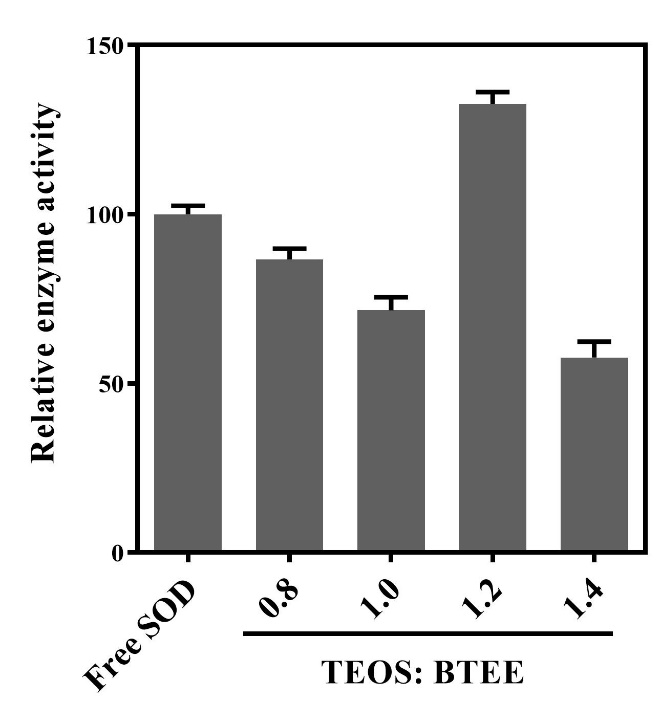
Fig. S1 SOD@MSN synthesized under various ligand ratios (****TEOS: BTEE from 0.8 to 1.4).**

**Fig. S2 Enzymatic activities of SOD@MSN under various ligand ratios.**

**
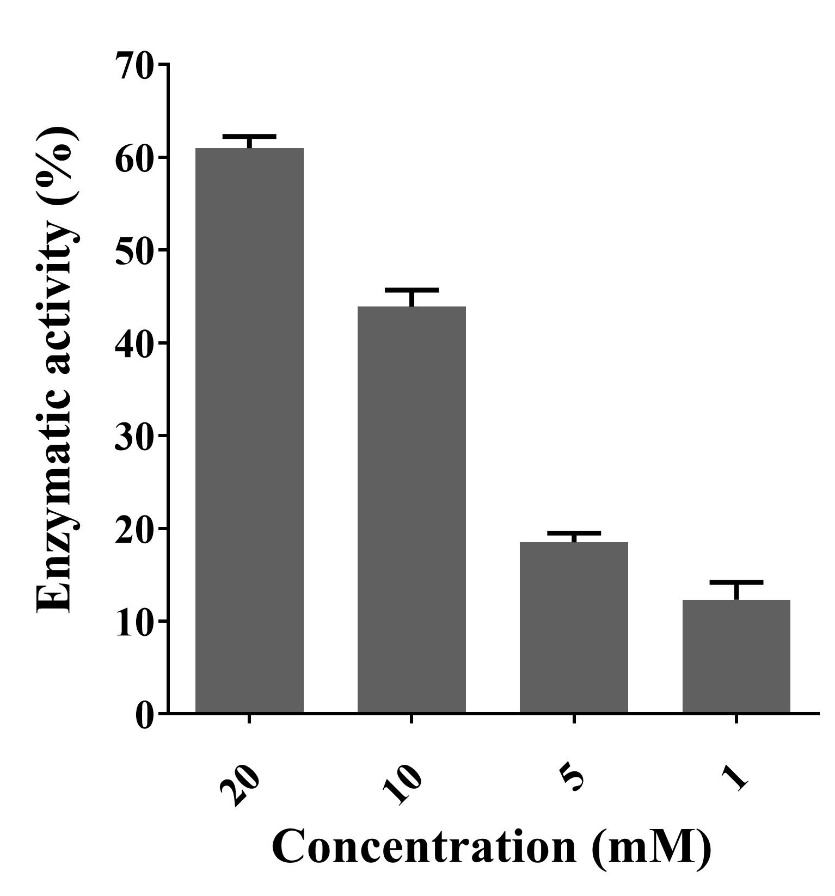
Fig. S3 SOD enzymatic activity of SOD@MSN detected in a concentration-dependent manner.**

**
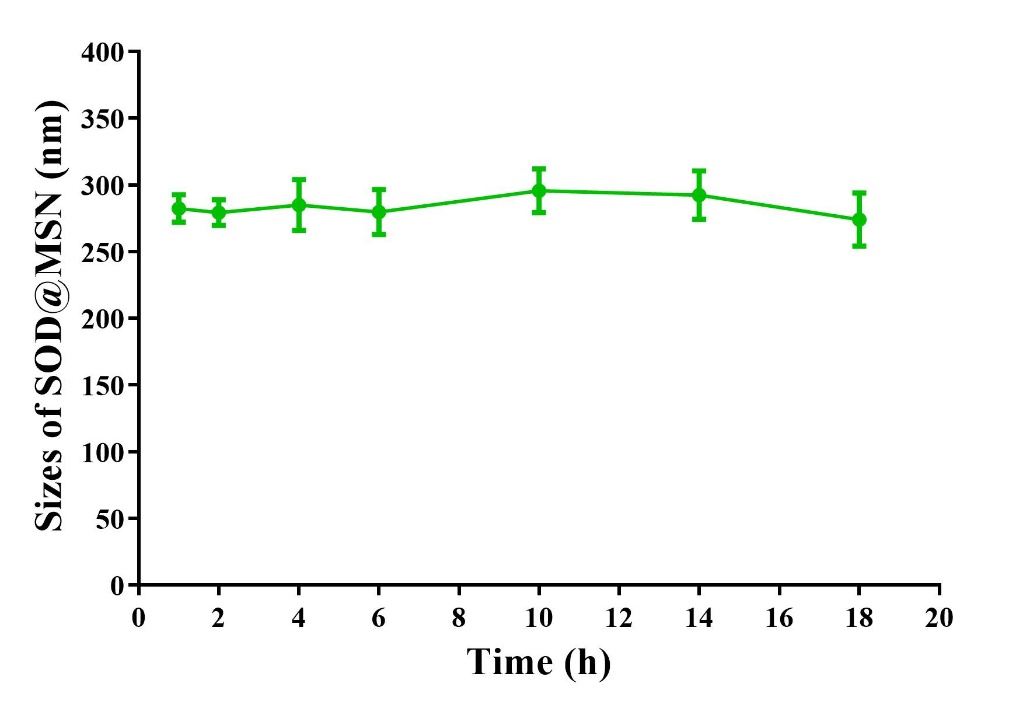
Fig. S4
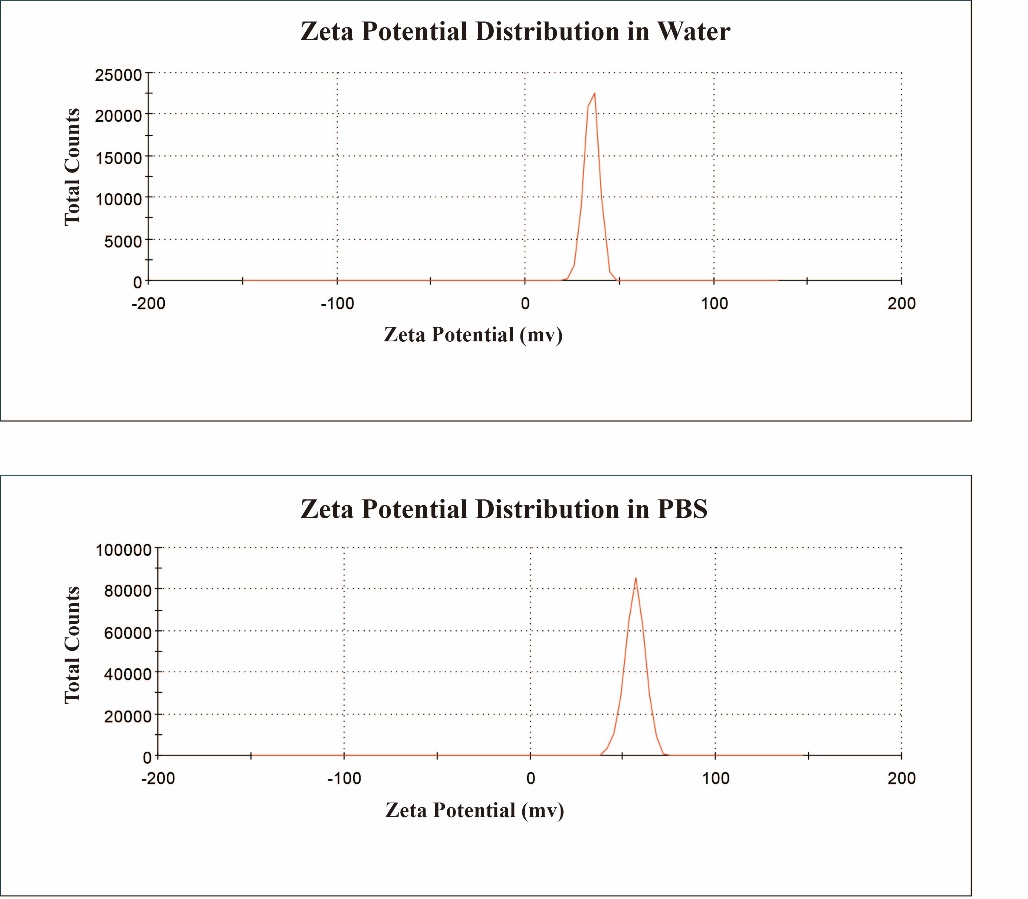
Zeta potential distribution of SOD@MSN in water and PBS solutions.**

**Fig. S5** **Sizes of SOD@MSN in the body of** **C. elegans detected in a time-dependent manner.**
